# Mechanism of Microbial Metabolite Leupeptin in the Treatment of COVID-19 by Traditional Chinese Medicine Herbs

**DOI:** 10.1128/mBio.02220-21

**Published:** 2021-09-28

**Authors:** Lifeng Fu, Shuai Shao, Yong Feng, Fei Ye, Xue Sun, Qingling Wang, Feng Yu, Qisheng Wang, Baoying Huang, Peihua Niu, Xuebing Li, Catherine C. L. Wong, Jianxun Qi, Wenjie Tan, George Fu Gao

**Affiliations:** a CAS Key Laboratory of Pathogen Microbiology and Immunology, Institute of Microbiology, Chinese Academy of Sciences, Beijing, China; b Center for Influenza Research and Early Warning (CASCIRE), CAS-TWAS Center of Excellence for Emerging Infectious Diseases (CEEID), Chinese Academy of Sciences, Beijing, China; c National Institute for Radiological Protection, China CDC, Beijing, China; d NHC Key Laboratory of Biosafety, National Institute for Viral Disease Control and Prevention, Chinese Center for Disease Control and Prevention, China CDC, Beijing, China; e Center for Precision Medicine Multi-Omics Research, Peking University Health Science Center, Peking University, Beijing, China; f Peking University First Hospital, Peking University, Beijing, China; g Shaanxi Natural Carbohydrate Resource Engineering Research Center, College of Food Science and Technology, Northwest University, Xi’an, China; h Shanghai Synchrotron Radiation Facility, Shanghai Advanced Research Institute, Chinese Academy of Sciences, Shanghai, China; i Peking-Tsinghua Center for Life Sciences, Peking University, Beijing, China; GSK Vaccines

**Keywords:** SARS-CoV-2, main protease, leupeptin, TCM modernization research, plants, microbiome, and microbial metabolites ecosystem

## Abstract

Coronavirus disease 2019 (COVID-19) has caused huge deaths and economic losses worldwide in the current pandemic. The main protease (M^pro^) of severe acute respiratory syndrome coronavirus 2 (SARS-CoV-2) is thought to be an ideal drug target for treating COVID-19. Leupeptin, a broad-spectrum covalent inhibitor of serine, cysteine, and threonine proteases, showed inhibitory activity against M^pro^, with a 50% inhibitory concentration (IC_50_) value of 127.2 μM *in vitro* in our study here. In addition, leupeptin can also inhibit SARS-CoV-2 in Vero cells, with 50% effective concentration (EC_50_) values of 42.34 μM. More importantly, various strains of streptomyces that have a broad symbiotic relationship with medicinal plants can produce leupeptin and leupeptin analogs to regulate autogenous proteases. Fingerprinting and structure elucidation using high-performance liquid chromatography (HPLC) and high-resolution mass spectrometry (HRMS), respectively, further proved that the Qing-Fei-Pai-Du (QFPD) decoction, a traditional Chinese medicine (TCM) formula for the effective treatment of COVID-19 during the period of the Wuhan outbreak, contains leupeptin. All these results indicate that leupeptin at least contributes to the antiviral activity of the QFPD decoction against SARS-CoV-2. This also reminds us to pay attention to the microbiomes in TCM herbs as streptomyces in the soil might produce leupeptin that will later infiltrate the medicinal plant. We propose that plants, microbiome, and microbial metabolites form an ecosystem for the effective components of TCM herbs.

## INTRODUCTION

Coronavirus disease 2019 (COVID-19) has caused a huge threat to global public health (34, 35). Moreover, the constant emergence of variants of concern (VOCs) of severe acute respiratory syndrome coronavirus 2 (SARS-CoV-2) may bring the risk of vaccine and antibody failure, such as VOC 501Y.V1 (α variant) or 501Y.V2 (β variant) (1). Thus, there is a demand to develop antiviral drugs. At present, many HIV and hepatitis C virus (HCV) drugs targeting the viral protease have been approved. SARS-CoV-2 can also produce two proteases, main protease (M^pro^) and papain-like protease (PL^pro^), which hydrolyze polyproteins 1a and 1ab for virus replication and maturation. M^pro^ cuts 11 sites of viral polyproteins 1a and 1ab to obtain 12 proteins, such as the nsp7-nsp8-nsp12 complex RNA-dependent RNA polymerase (RdRp), nsp13 helicase-triphosphatase, and nsp14-nsp10 complex *S*-adenosyl methionine (SAM)-dependent (guanine-N7) methyltransferase (2). Therefore, M^pro^ is thought to be an ideal target for treating COVID-19.

**FIG 1:**
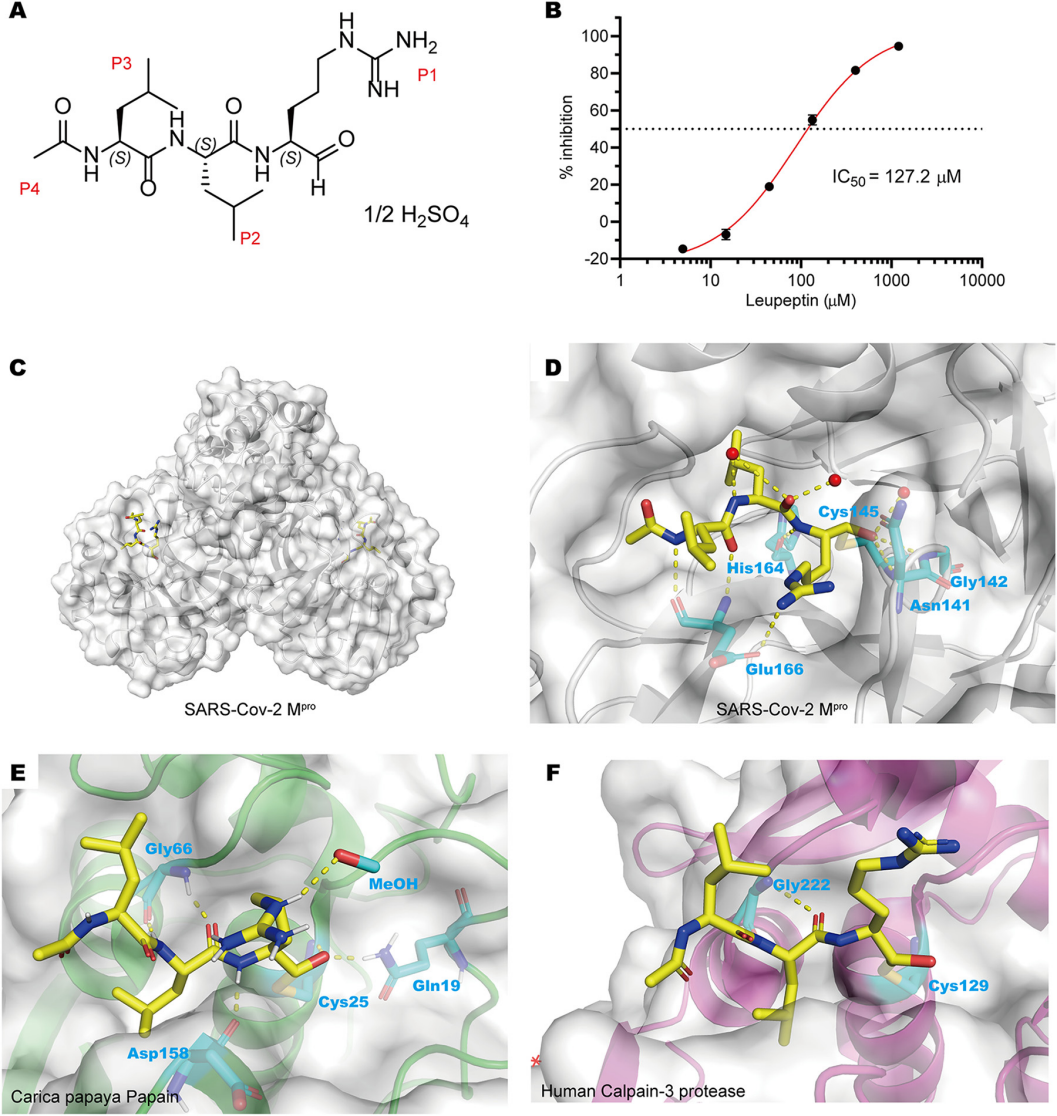
Leupeptin can inhibit the catalytic activity of SARS-CoV-2 M^pro^ *in vitro*. (A) Chemical structure of leupeptin hemisulfate. (B) Inhibitory curve of leupeptin hemisulfate against SARS-CoV-2 M^pro^. (C) Overall structure of SARS-CoV-2 M^pro^ dimer in complex with leupeptin. (D) Detailed interactions between leupeptin (yellow) and SARS-CoV-2 M^pro^. (E) Binding pocket of leupeptin bound to Carica papaya papain protease (PDB accession number 1POP). (F) Binding pocket of leupeptin bound to human calpain-3 protease (PDB accession number 6BKJ).

Leupeptin (Fig. 1A) is a secondary metabolite produced by actinomycetes (3–6), which has broad-spectrum protease-inhibitory activities, including serine, cysteine, and threonine proteases (7–11). Leupeptin treatment can prevent or delay muscular dystrophy in mouse or chicken models (12). Rabbits treated with leupeptin did not develop experimental autoimmune myasthenia gravis despite repeated acetylcholine receptor injections (13). These studies showed that leupeptin has a certain clinical value. In addition, human coronavirus strain 229E can be inhibited by leupeptin in plaque reduction tests (14). Here, we found that leupeptin could also inhibit M^pro^ of SARS-CoV-2. Interestingly, high-performance liquid chromatography (HPLC) fingerprinting and high-resolution mass spectrometry (HRMS) showed that the Qing-Fei-Pai-Du (QFPD) decoction, a traditional Chinese medicine (TCM) formula, contains leupeptin. These results reveal that leupeptin may contribute to the QFPD decoction in the treatment of COVID-19, which had been popularly used in Wuhan in the first wave of the outbreak.

## RESULTS AND DISCUSSION

### SARS-CoV-2 M^pro^-inhibitory activity.

In order to verify whether leupeptin has inhibitory activity against M^pro^, we expressed and purified M^pro^ of SARS-CoV-2 according to our previous work (15, 16). Fluorescence resonance energy transfer (FRET) enzymatic assays showed that leupeptin has some inhibitory activity against M^pro^, with a 50% inhibitory concentration (IC_50_) value of 127.2 μM (Fig. 1B). However, the progress curve of substrate hydrolysis did not indicate a clear time-dependent inhibition of leupeptin against M^pro^ (see Fig. S1 in the supplemental material). Therefore, whether leupeptin is a covalent inhibitor of M^pro^ still needs to be further evaluated, which is addressed in the following experiments.

10.1128/mBio.02220-21.1 FIG S1 Progress curve of peptide hydrolysis by SARS-CoV-2 M^pro^ in the presence of leupeptin. Download FIG S1, TIF file, 0.4 MB. Copyright © 2021 Fu et al. 2021 Fu et al. https://creativecommons.org/licenses/by/4.0/ This content is distributed under the terms of the Creative Commons Attribution 4.0 International license.

### Inhibitory mechanisms of leupeptin.

We further determined the crystal structures of M^pro^-leupeptin at resolutions of 1.7 Å to figure out the inhibitory mechanisms of leupeptin (Fig. 1C and Table S1). A room-temperature crystal structure of M^pro^-leupeptin at a lower resolution of 2.2 Å has also been solved (17), and during the preparation of the manuscript, a similar complex structure, also at a 1.7-Å resolution, was reported (18). We found that the mode of binding of leupeptin with M^pro^ was almost the same in these three structures except for the conformation of the P1 guanidine residue. The unambiguous electron density maps show that leupeptin binds in the active site of M^pro^ (Fig. S2). The mode of binding of leupeptin with M^pro^ is different from that of leupeptin with other cysteine proteases, such as papain (Fig. 1E) or human calpain-3 (Fig. 1F). In the complex structure, the Sγ atom of the nucleophilic Cys145 in M^pro^ forms a C-S covalent bond with the aldehyde carbon of leupeptin (Fig. 1D and Fig. S2). The hemiacetal oxygen of leupeptin faces the “oxyanion hole” with the formation of two hydrogen bonds with the main-chain amides of Cys145, Asn141, and Gly142, which is similar to the trypsin-leupeptin complex (11) (Fig. 1D). But this result is inconsistent with the results of the substrate hydrolysis progress curve. The reason for this may be that the aldehyde group of leupeptin can reversibly react with Cys145 of M^pro^ by an attack of water or other nucleophiles. The negatively charged guanidine residue can be tolerated by the S1 subsite and form one hydrogen-bonding interaction with Glu166. The backbone of leupeptin forms two other hydrogen-bonding interactions with Glu166 and His164 (Fig. 1D). The hydrophobic P2 isobutyl (i-Bu) group of leupeptin can fit into the hydrophobic S2 subsite well. On the contrary, the hydrophobic P3 i-Bu group was exposed to the solvent, which seems to have no interactions with the surrounding amino acids of the S3 subsite. In addition, the P4 acetyl residue cannot fully occupy the S4 subsite. All of these factors are responsible for the relatively weak inhibitory activity.

10.1128/mBio.02220-21.2 FIG S2 Electron density of SARS-CoV-2 M^pro^ complexed with leupeptin. The panels show portions of 2*F*_o_-*F*_c_ electron density maps for leupeptin contoured at 1.0 sigma. Download FIG S2, JPG file, 1.0 MB. Copyright © 2021 Fu et al. 2021 Fu et al. https://creativecommons.org/licenses/by/4.0/ This content is distributed under the terms of the Creative Commons Attribution 4.0 International license.

10.1128/mBio.02220-21.4 TABLE S1 Diffraction data and refinement statistics. Download Table S1, DOCX file, 0.02 MB. Copyright © 2021 Fu et al. 2021 Fu et al. https://creativecommons.org/licenses/by/4.0/ This content is distributed under the terms of the Creative Commons Attribution 4.0 International license.

### Antiviral potency of leupeptin against SARS-CoV-2.

**FIG 2:**
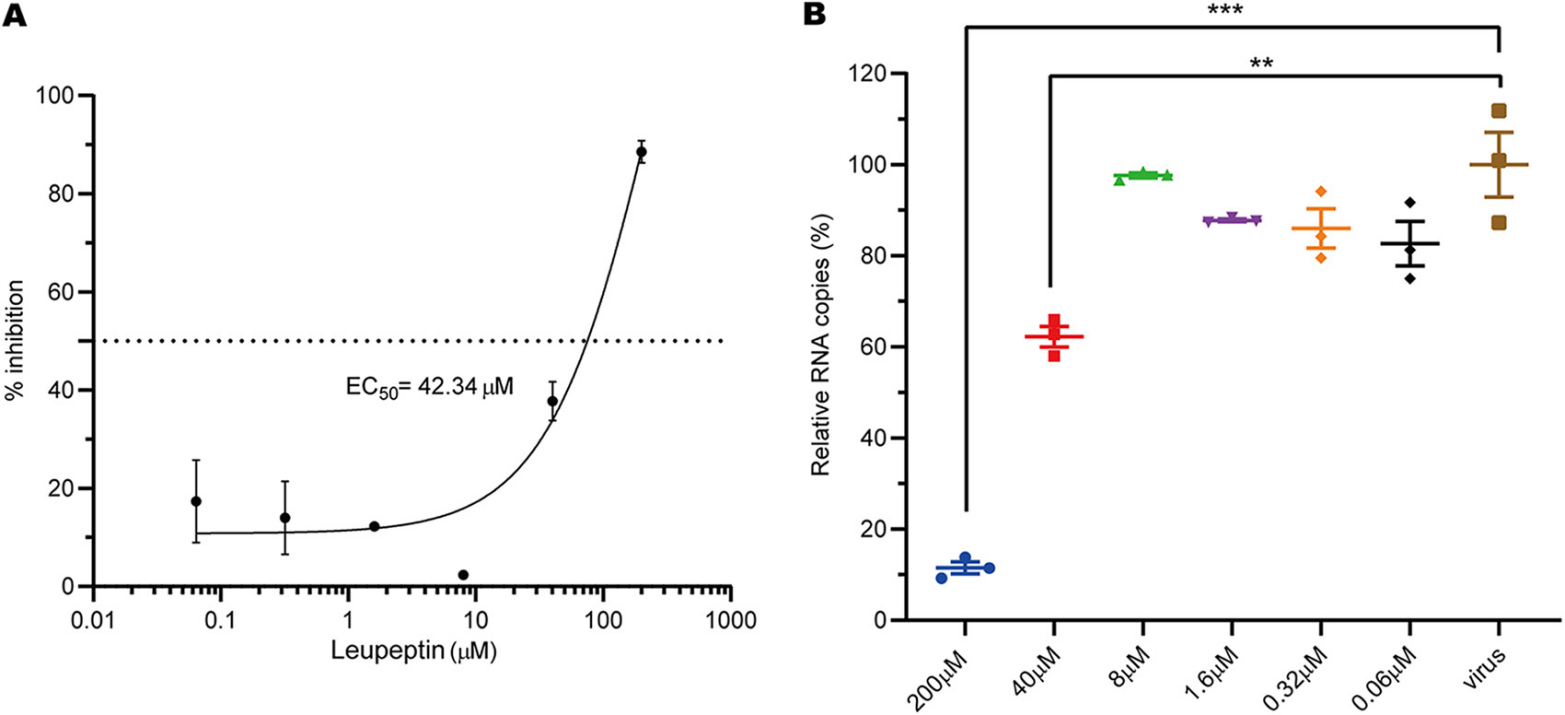
Inhibition of SARS-CoV-2 replication in Vero cells by leupeptin. (A) Inhibitory curve of leupeptin against SARS-CoV-2 replication. (B) Relative concentration of vRNA present in the supernatant at 72 h postinfection determined by reverse transcription-quantitative PCR (qRT-PCR) analysis after leupeptin treatment. Continued *t* test *P* values indicate statistical significance (**, *P* < 0.01; ***, *P* < 0.001).

The IC_50_ value of leupeptin in plaque reduction tests was 0.4 μg/ml (about 1 μM) against human coronavirus strain 229E (14). Combined with the SARS-CoV-2 M^pro^ activity inhibition results, this indicates that leupeptin may inhibit the replication of SARS-CoV-2 in cells. Subsequent antiviral assays showed that the RNA levels of SARS-CoV-2 can be inhibited by leupeptin in Vero cells, with 50% effective concentration (EC_50_) values of 42.34 μM (Fig. 2A). Moreover, viral RNA (vRNA) copy numbers dropped significantly in the presence of 40 μM leupeptin (Fig. 2B). Leupeptin can inhibit human coronavirus strain 229E better than SARS-CoV-2 in cells. The possible reason for this is that leupeptin inhibits trypsin and cathepsin L involved in the 229E virus entry process (19). However, the entry of SARS-CoV-2 needs TMPRSS2 and furin-like proteases instead of cathepsin (20).

### HPLC fingerprinting and HRMS analysis.

Leupeptin and leupeptin analogs were isolated from various strains of *Streptomyces* spp., such as Streptomyces roseus MA839-A1 (5), Streptomyces exfoliatus SMF13 (4), Streptomyces griseus strain 254 (3), and Streptomyces lavendulae (21). Autogenous protease inhibitors can regulate cellular turnover metabolism associated with secondary metabolism and morphogenesis by inhibiting the activity of the protease (22). S. roseus strains have been isolated from rhizospheric soil samples under cultivation in Curcuma longa L (23). *S. griseus* strains have been isolated from many habitats, including soils, marine sediments, and medicinal plants such as Kandelia candel (24). *S. lavendulae* strains have also been isolated from soils and Ginkgo biloba (25). *Streptomyces* accounts for 60% of endophytic actinobacteria isolated from Glycyrrhiza inflata Batal (26). In general, *Streptomyces* and medicinal plants have a broad symbiotic relationship. On the other hand, TCM composed of medicinal plants showed potential for the treatment of COVID-19 (27, 28). Thus, we suspected that some TCM formulas for the treatment of COVID-19 may contain leupeptin. The QFPD decoction, a TCM formula, has shown therapeutic effects on mild, ordinary, and severe COVID-19 patients (29). Early treatment with QFPD was associated with the recovery of patients with COVID-19, including faster recovery, a shorter time to viral shedding, and a shorter duration of hospital stay. The overall response rates for QFPD were over 90% among 782 cases in the clinic (30). This TCM formulation was selected as one of the recommended therapeutic regimens by the National Administration of Traditional Chinese Medicine (NATCM) and is widely used in China (31, 32). QFPD contains 20 kinds of herbs and a kind of mineral, which is mainly derived from 4 different classical prescriptions in the ancient work *Shang Han Lun*.

**FIG 3:**
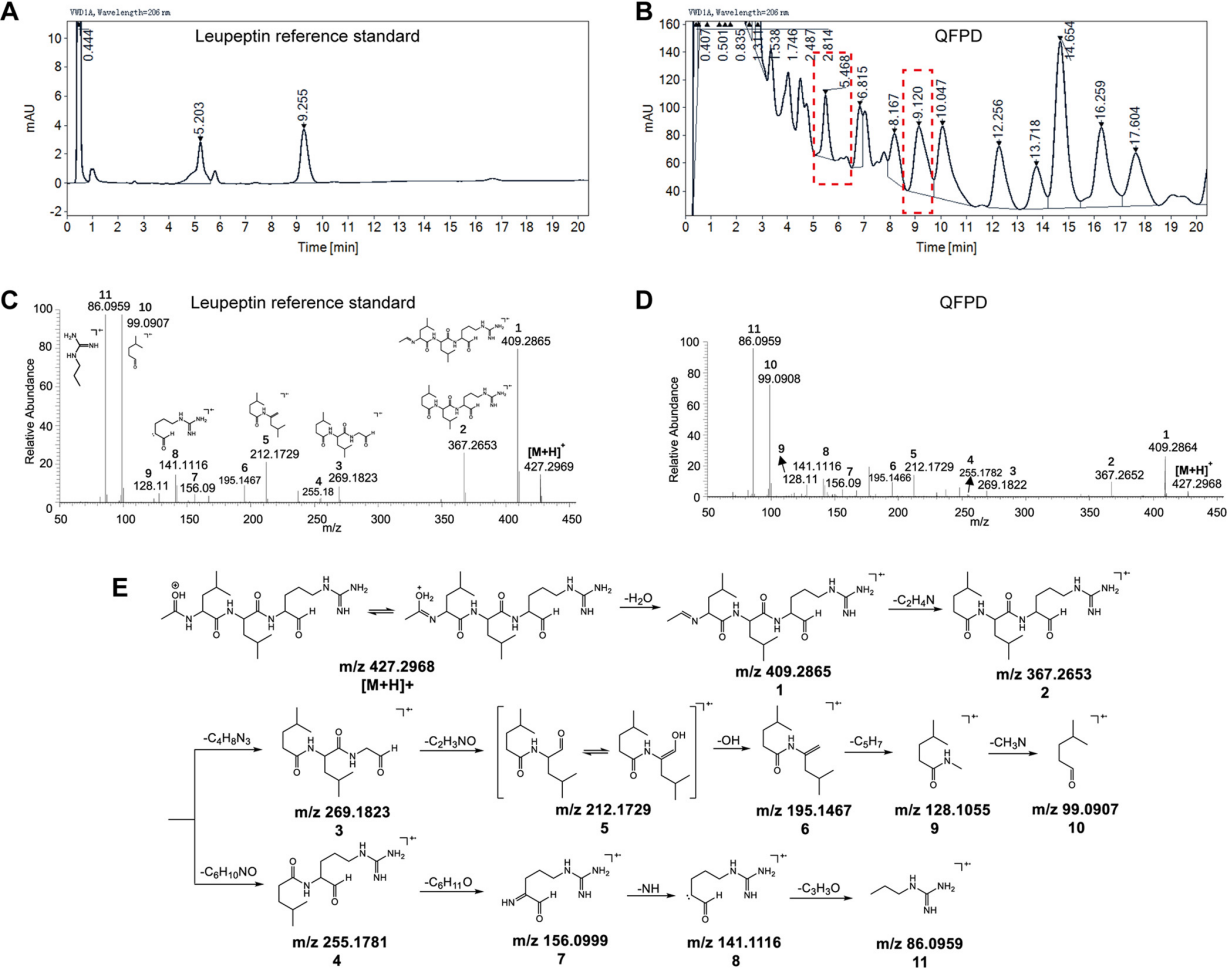
HPLC and HRMS analyses of leupeptin and the QFPD decoction. (A) HPLC chromatogram of the leupeptin reference standard. (B) HPLC chromatogram of the QFPD decoction. The chromatographic peaks in boxes with dotted lines indicate the probable leupeptin substance in the QFPD decoction. (C) MS/MS spectrum of the precursor ion (*m/z* 427.2969) in the leupeptin reference standard. (D) MS/MS spectrum of the precursor ion (*m/z* 427.2968) in the QFPD decoction. (E) Proposed fragmentation pathway of leupeptin. The numbers 1 to 11 represent the different fragments of leupeptin.

To characterize whether leupeptin is one of the substances of QFPD, we applied HPLC and HRMS to both the leupeptin reference standard and the QFPD decoction. Chromatogram of the leupeptin reference standard with two retention times, 5.203 and 9.255 min, at a 206-nm UV absorption, caused by the tautomeric isomers (33), is exhibited in Fig. 3A. Corresponding chromatographic peaks at retention times of 5.468 and 9.120 min were also found by chromatographic fingerprinting of the QFPD aqueous solution (Fig. 3B). HRMS was used for the further structure elucidation of leupeptin in QFPD as well as the reference standard. The observed protonated precursor ion [M + H]^+^ of the leupeptin reference standard was *m/z* 427.2969 (Fig. 3C), while that of the QFPD solution was *m/z* 427.2968 (Fig. 3D). The mass difference was 0.2 ppm, which shows that the precursor ions were likely identical. Moreover, the major fragment ions shown in the tandem mass spectrometry (MS/MS) spectrum of the QFPD decoction highly correspond to those of the reference standard (Fig. 3C to E and Fig. S3). These results confirm that leupeptin is one of the substances in the QFPD decoction.

10.1128/mBio.02220-21.3 FIG S3 Mirror plot of the MS/MS spectrum of *m/z* 427.2968 extracted from QFPD (top) and the standard leupeptin (*m/z* 427.2969) (bottom). Mass error tolerances of the precursor and fragments were <0.2 ppm (±0.1 ppm). The coincidence score of these two spectra was calculated as 94.2%. Download FIG S3, JPG file, 0.2 MB. Copyright © 2021 Fu et al. 2021 Fu et al. https://creativecommons.org/licenses/by/4.0/ This content is distributed under the terms of the Creative Commons Attribution 4.0 International license.

**FIG 4:**
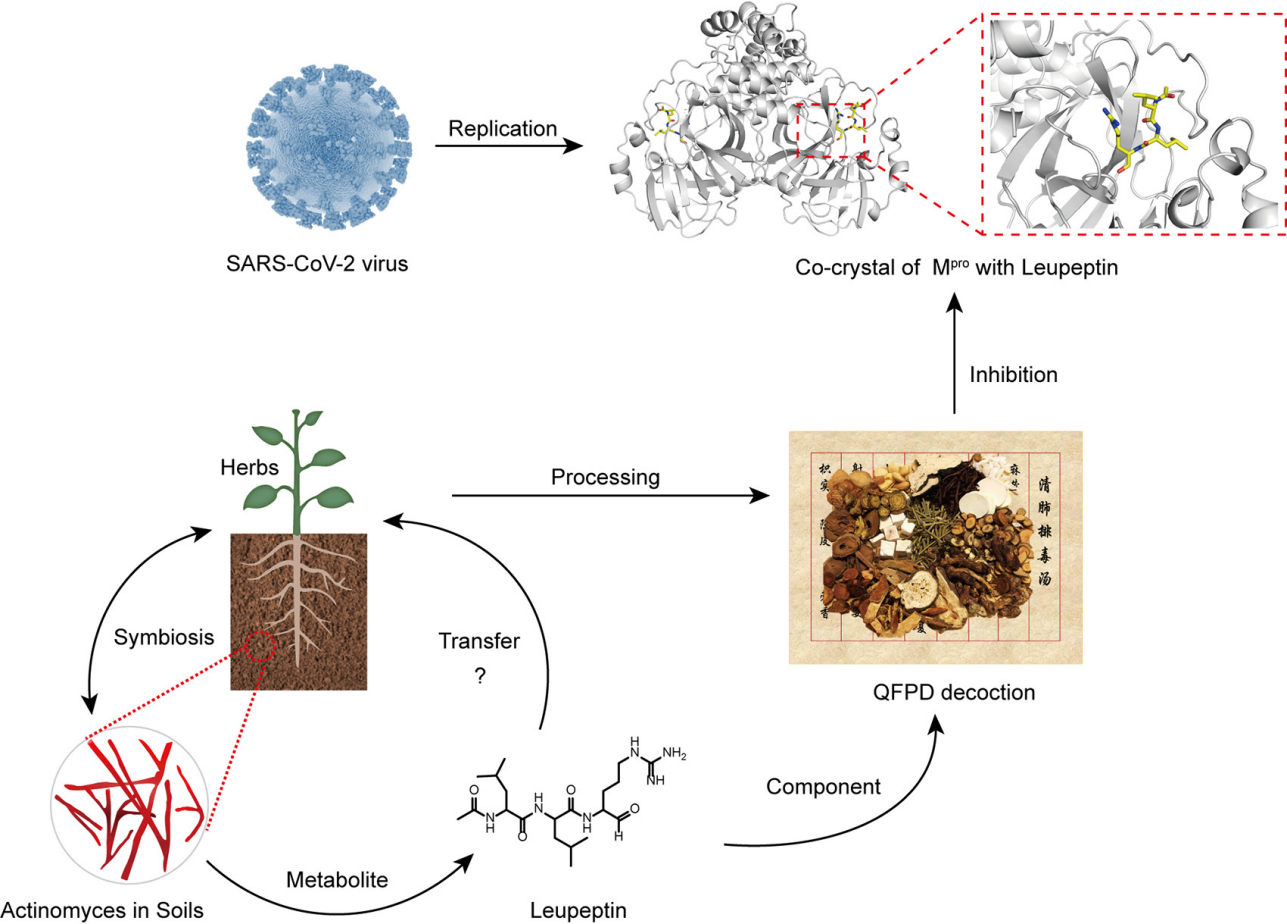
Plant-symbiotic actinomyces and antiviral activity ecology of microbial bioactive compounds, such as leupeptin, contribute to the antiviral activity of the QFPD decoction against SARS-CoV-2 M^pro^.

In a word, we have identified leupeptin, a metabolite produced by PSA, which showed antiviral activity in both enzyme assays and cell culture. Moreover, we identified that leupeptin is one of the substances in the QFPD decoction and well characterized by HPLC fingerprinting and HRMS. These results suggest that leupeptin likely contributes to the antiviral activity of the QFPD decoction against SARS-CoV-2. Also, the PSA metabolite leupeptin can transfer from microbes to medicinal plants (Fig. 4). Thus, this reminds us to take the role of microbiomes in TCM modernization seriously. Our study has opened a new door for the effective assessment of TCM formulas by evaluating the plants, microbiome, and microbial metabolites ecosystem.

## MATERIALS AND METHODS

### Protein expression and purification.

The expression and purification of the native SARS-CoV-2 M^pro^ protein were performed according to the literature (15). In brief, recombinant proteins with His and SUMO tags were expressed in Escherichia coli strain BL21(DE3) as soluble proteins after induction with 0.5 mM isopropyl-β-d-thiogalactopyranoside (IPTG) at an optical density at 600 nm (OD_600_) of 0.6 to 0.8 and expression at 16°C for 18 h. The cells were lysed by sonication in lysis buffer (20 mM Tris, 50 mM NaCl [pH 8.0]). After centrifugation at 20,133 × *g* for 30 min, the supernatants were then purified by affinity chromatography using HisTrap HP 5-ml columns (GE Healthcare). The target protein was eluted with elution buffer (20 mM Tris, 50 mM NaCl, 300 mM imidazole [pH 8.0]). The purified proteins were incubated with SUMO protease (catalog number P2312M; Beyotime) at 30°C for 2 h after centrifugation according to the manufacturer’s instructions. The hydrolyzed proteins were then purified by affinity chromatography using HisTrap HP 5-ml columns (GE Healthcare) again and further purified by size exclusion chromatography using a Hiload 16/600 Superdex 75-pg column (GE Healthcare) equilibrated with binding buffer (10 mM Tris-HCl, 1 mM dithiothreitol [DTT], 1 mM EDTA [pH 7.5]).

### M^pro^ enzyme activity inhibition test.

A total of 20 mM leupeptin hemisulfate (catalog number T6564; TargetMol) in deionized water was diluted to 2 mM to 31.25 μM with 25 mM Tris buffer (pH 8.0). A 30-μl inhibitor solution with a series of concentrations in 25 mM Tris buffer (pH 8.0) was first mixed with 10 μl 100 μM peptide substrate (Dabcyl-TSAVLQ↓SGFRKMK-Edans; GenScript). Next, 10 μl of a final concentration of 200 nM M^pro^ was added to the plate. The relative fluorescence unit (RFU) value was measured with an excitation wavelength of 360 nm and an emission wavelength of 490 nm at 37°C for 1 h by using a SpectraMax Paradigm multimode detection platform (Molecular Devices, USA). Experiments were performed in triplicate. The enzyme activity reaction rate and inhibition rate were calculated by using MS Excel. The inhibition curve was plotted by using GraphPad Prism 8.0.

### Crystallization.

Concentrations of 8 mg/ml and 12 mg/ml M^pro^ (in a solution containing 10 mM Tris, 1 mM EDTA, and 1 mM DTT [pH 7.5]) were incubated with 20 mM leupeptin at 4°C for 18 h. All the crystals were obtained by using the sitting-drop vapor diffusion method with 1 μl of protein mixed with 1 μl of a reservoir solution and then equilibrating the mixture against 100 μl of the reservoir solution at 18°C. The initial crystallization screenings were carried out using commercially available kits. The M^pro^-leupeptin complexes were crystallized in a solution containing 20% polyethylene glycol 1500 (PEG 1500) and 0.1 M Bis-Tris (pH 6.5).

### Data collection and structure determination.

Diffraction data were collected at Shanghai Synchrotron Radiation Facility (SSRF) BL17U (wavelength, 0.97918 Å). For data collection, the crystals were cryoprotected by briefly soaking them in reservoir solution supplemented with 20% (vol/vol) glycerol before flash-cooling in liquid nitrogen. The data set was processed with HKL2000 software. The native M^pro^ complex structure was determined by the molecular replacement method using Phaser with the previously reported SARS-CoV-2 M^pro^ structure (PDB accession number 7C6S). The atomic models were completed with Coot and refined with phenix.refine in Phenix, and the stereochemical qualities of the final models were assessed with MolProbity. Data collection, processing, and refinement statistics are summarized in Table S1 in the supplemental material. All structural figures were generated using PyMOL software (http://www.pymol.org).

### Cells and viruses.

African green monkey kidney Vero cells were maintained in Dulbecco’s modified Eagle’s medium (DMEM) (Gibco, USA) supplemented with 10% fetal bovine serum (FBS) (Gibco, USA), 200 mg/ml streptomycin, and 200 IU/ml penicillin at 37°C. COVID-19 virus was isolated from the Wuhan seafood market by the China CDC. All the infection experiments were performed in a biosafety level 3 (BLS-3) laboratory.

### *In vitro* antiviral assays.

A total of 20 mM leupeptin hemisulfate (catalog number T6564; TargetMol) in deionized water was diluted to 200 μM to 0.06 μM with DMEM containing 1% FBS. Vero cells cultured overnight in 96-well plates were infected by virus at a multiplicity of infection (MOI) of 0.01 for 2 h. The medium was removed, and fresh drug-containing medium was then added to the cells. After 48 h, the cells were lysed in lysis buffer. The viral RNA in 100 μl of the cell supernatant was quantified by reverse transcription-PCR (RT-PCR). Seventy-two hours later, the changes of cytopathic effect were also observed by microscopy. Experiments were performed in triplicate. The experimental results were processed using MS Excel and GraphPad Prism 8.0.

### HPLC analysis.

A total of 0.1 g/ml of QFPD powder (catalog number JZT-QFPDT-0318-PG-0321; Jointown Pharmaceutical Group, Wuhan, China) and 1 μg/ml leupeptin (catalog number L5793; Sigma-Aldrich, St. Louis, MO, USA) in ultrapure water were prepared. Chromatographic fingerprinting analysis of leupeptin and QFPD was performed by using an HPLC system (Agilent 1260 Infinity II Prime). The column was a Zorbax Eclipse Plus 95-Å C_18_ HPLC column (2.1 by 150 mm, 3.5 μm) (catalog number 959763-902; Agilent, CA, USA). The solvent system consisted of 0.1% (vol/vol) phosphoric acid in MilliQ water as component A, while component B was acetonitrile and was run at a flow rate of 1 ml/min. The solvent gradient was begun with 10% B for 0 to 10 min, 10% to 30% B for 10 to 85 min, and 30 to 50% B for 85 to 110 min, followed by an equilibration time of 10 min with 10% B. The temperature of the column was 40°C, and the target tripeptide was successfully detected at a wavelength of 206 nm (the maximum UV absorption wavelength of leupeptin). Injection volumes of 10 μl of each sample and the standards were used for the HPLC analysis, and the supernatant liquid of the QFPD sample solution and the leupeptin standard solution was obtained by centrifugation at 20,133 × *g*.

### High-resolution mass spectrometry analysis.

MS-grade methanol (MeOH) was purchased from Merck (Darmstadt, Germany). Approximately 0.1 g QFPD powder was added to 1.0 ml MeOH to extract leupeptin. The complex was then vortexed for 30 s and sonicated for 30 min, followed by 20 min of centrifugation at 16,000 × *g* at 4°C. The extractant and 500 ng/ml of the leupeptin solution were directly infused into an electrospray ionization (ESI)-Q-orbitrap MS instrument (Thermo Q-Exactive Plus) at a flow rate of 10 μl/min for leupeptin identification. The analytes, which were monitored under a targeted data-dependent MS2 (ddMS2) mode with positive ionization, were ionized with a vaporizing temperature of 350°C and a spray voltage of 3,800 V. The MS/MS analysis was conducted with a collision energy of 35 V, a resolution of 70,000 full width at half maximum (FWHM), an automatic gain control (AGC) target of 5e4, a maximum inject time of 100 ms, and an isolation window of *m/z* 2.0. Xcalibur 4.1.50 software was employed to acquire and process MS and MS/MS data.

### Data availability.

Coordinates and structure factors have been deposited in the Protein Data Bank under accession number 7EIN.
