# Supplementary figures and images for: Mechanism of Microbial Metabolite Leupeptin in the Treatment of COVID-19 by Traditional Chinese Medicine Herbs
Source: mBio. 2021 Sep 28;12(5):e02220-21. doi: 10.1128/mBio.02220-21 (PMC8546846; doi:10.1128/mBio.02220-21)

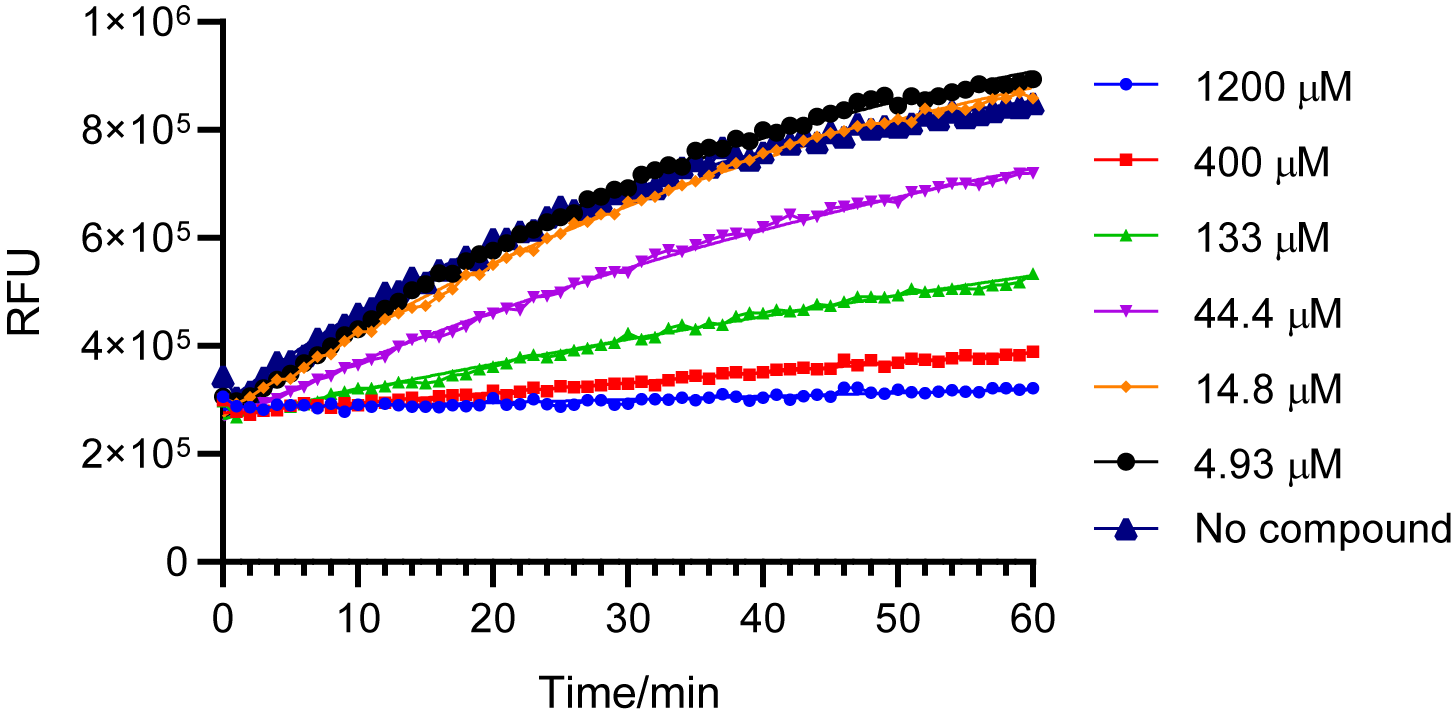

Supplement: FIG S1 [file mbio.02220-21-sf001.tif]

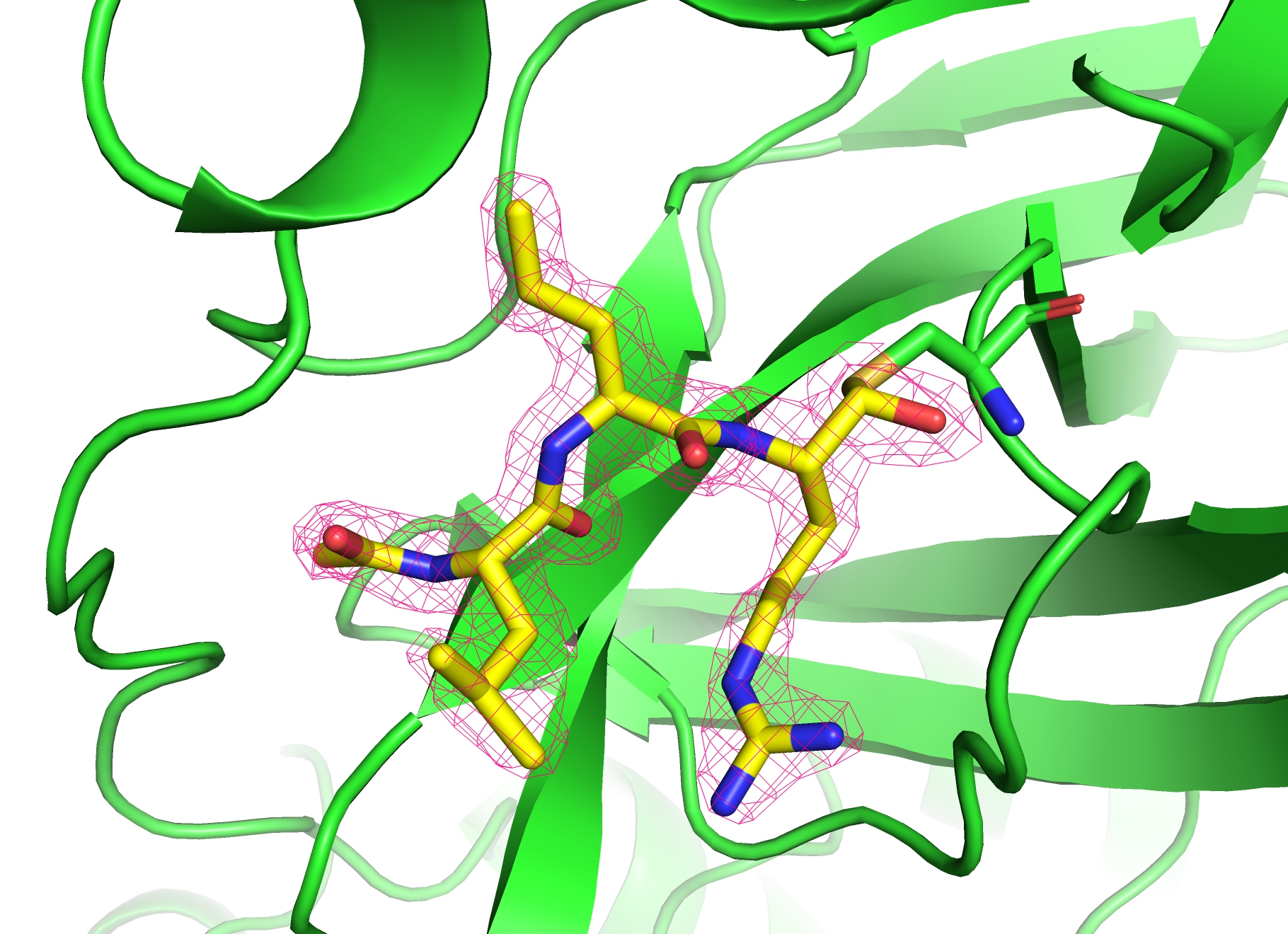

Supplement: FIG S2 [file mbio.02220-21-sf002.jpg]

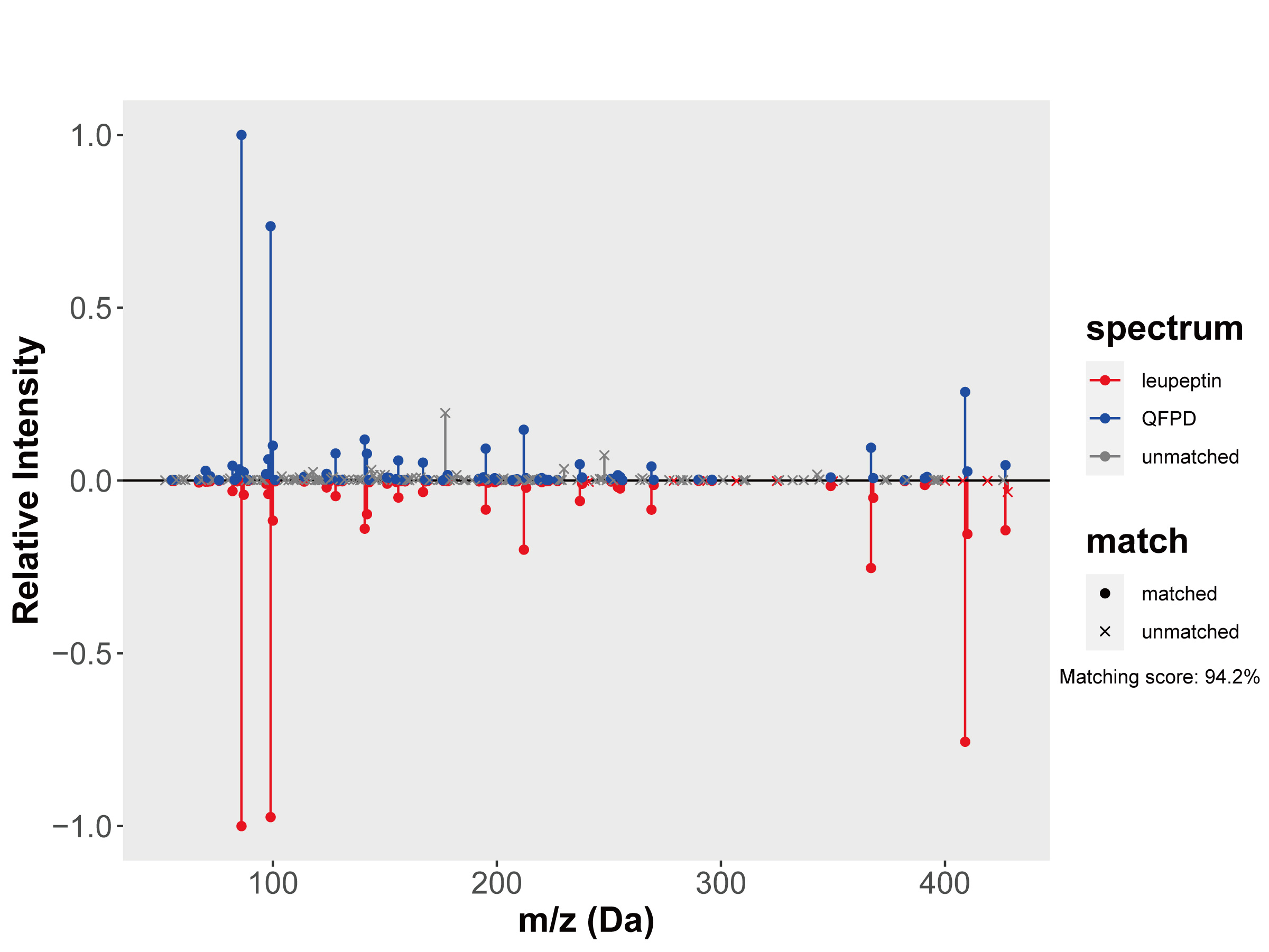

Supplement: FIG S3 [file mbio.02220-21-sf003.jpg]
