# Supplementary material for: Mechanism of Microbial Metabolite Leupeptin in the Treatment of COVID-19 by Traditional Chinese Medicine Herbs
Source: mBio. 2021 Sep 28;12(5):e02220-21. doi: 10.1128/mBio.02220-21 (PMC8546846; doi:10.1128/mBio.02220-21)
Supplement: TABLE S1 [file mbio.02220-21-st001.docx]

**TABLE S1. Diffraction data and refinement statistics***

|  | M^pro^-leupeptin |
| --- | --- |
| **PDB Code** | 7EIN |
| **Data collection** |  |
| Space group | P21 |
| Cell dimensions |  |
| *a*, *b*, *c* (Å) | 46.04, 53.13, 113.64 |
| α, β, γ (°)  Wavelength (Å) | 90, 101.05, 90  0.979 |
| Resolution (Å) | 50-1.70 (1.76-1.70) |
| *R*_merge_ | 0.063 (1.051) |
| *I* / σ*I* | 29.9 (2.3) |
| CC1/2 | 0.999 (0.867) |
| Completeness (%) | 100.0 (100.0) |
| Redundancy | 6.8 (6.9) |
|  |  |
| **Refinement** |  |
| Resolution (Å) | 47.96 - 1.70 |
| No. reflections | 59085 |
| *R*_work_ / *R*_free_ | 0.2350/0.2602 |
| No. atoms |  |
| Protein | 4632 |
| Ligand/ion | 62 |
| Water | 534 |
| *B*-factors |  |
| Protein | 18.9 |
| Ligand/ion | 22.9 |
| Water | 28.4 |
| R.m.s. deviations |  |
| Bond lengths (Å) | 0.009 |
| Bond angles (°) | 0.796 |
| Ramachandran plot |  |
| Favored (%) | 98.49 |
| Allowed (%) | 1.17 |
| Outliers (%) | 0.34 |

*Values in parentheses are for highest-resolution shell.
